# Improving the estimation of environment parameters via a two-qubit scheme

**DOI:** 10.1038/s41598-024-57150-7

**Published:** 2024-03-21

**Authors:** Ali Raza Mirza, Adam Zaman Chaudhry

**Affiliations:** 1 School of Science and Engineering, Lahore University of Management Sciences (LUMS), Opposite Sector U, D.H.A, Lahore, 54792 Pakistan; 2 Department of Physics, University of Surrey, Guildford, GU2 7XH United Kingdom

**Keywords:** Quantum physics, Quantum information, Quantum metrology

## Abstract

We demonstrate how using two qubits can drastically improve the estimation of environment parameters compared to using only a single qubit. The two qubits are coupled to a common harmonic oscillator environment, and the properties of the environment are imprinted upon the dynamics of the two qubits. The reduced density matrix of only one of these qubits contains a decoherence factor and an additional factor taking into account the indirect interaction induced between the qubits due to the interaction with their common environment. This additional factor can drastically improve the estimation of the environment parameters, as quantified by the quantum Fisher information. In particular, we investigate the estimation of the cutoff frequency, the coupling strength, and the temperature using our two-qubit scheme compared to simply using a single qubit. We find that the precision of the estimates can be improved by orders of magnitude.

## Introduction

Open quantum systems have attracted great interest over the past few decades as they have an important role in developing modern quantum technologies^1,2^. To understand quantum dynamics properly, the effect of the environment must be taken into account as every quantum system inevitably interacts with its environment, leading to decoherence^3,4^. Analyzing the effect of decoherence entails knowing parameters such as the system-environment coupling strength and the environment temperature. One useful method to measure these parameters is to consider a quantum probe (a small and controllable quantum system) interacting with its environment^5–22^. Once the dynamics of the probe are obtained, measurements on the probe allow us to estimate several properties associated with the environment. The precision of these estimates is encapsulated in the quantum Fisher information (QFI)^23–32^. To minimize the error in the estimates, as dictated by the Cramer–Rao bound, one needs to maximize the QFI.

To date, many efforts have been put forward to estimate the environment parameters by using single-qubit and two-qubit quantum probes^8,14,20,21,33^. In particular, the focus has been on estimating the parameters of a harmonic oscillator environment via a single-qubit probe, taking the initial probe-environment state to be a simple product state. Significantly, it has been argued that, compared to a single-qubit probe, using a two-qubit probe is largely not beneficial^14^. In this paper, we argue on the contrary by using a different scheme. Rather than performing measurements on two qubits to deduce the environment parameters, we show that the QFI can be drastically increased if we couple two qubits to their common environment but perform measurements on only one of them. In other words, we trace out one of the qubits. The common environment induces an indirect interaction between the two qubits comprising the probe, and it is this indirect interaction, which depends on the environment parameters, that leads to the increase in the QFI. Moreover, if the environment is such that the probe-environment interaction is strong, then the initial probe-environment correlations can become important^34–65^. In this regard, it has been recently shown that these initial probe-environment correlations can also drastically increase the QFI^66^. In this spirit, we also take the initial correlations into account to show that, besides the indirect interaction, the initial correlations can further increase the QFI. Note that while we trace out one qubit, we still consider our probe to be a two-qubit quantum probe, as the presence of the second qubit is essential to increase the QFI drastically.

We start the presentation of our scheme by analyzing the dynamics of the two-qubit quantum probe interacting with a common harmonic oscillator environment. Assuming relaxation timescales are much longer than the dephasing timescales, we can ignore dissipation effects; that is, we consider pure dephasing. We allow our system and the environment to interact until they achieve a joint thermal equilibrium state. A projective measurement is then performed on the probe to prepare the initial probe state. After that, the two-qubit probe interacts with the environment. We then perform a partial trace over the environment and the second qubit to obtain a $2\times 2$ density matrix describing the dynamics of one qubit only. This density matrix contains the effect of decoherence, the initial correlations, and the indirect interaction between the two qubits. With this density matrix obtained, we work out the QFI, which is obviously a function of the interaction time between the probe and the environment. The idea is to choose this interaction time to maximize the QFI. We conclusively show that the corresponding maximum QFI can be surprisingly much greater than the QFI obtained with a single-qubit probe. We emphasize that this is not simply an increase in the QFI via a simple scaling factor (such as a doubling of the QFI); rather, the behavior of the QFI qualitatively changes due to the presence of the indirect interaction.

## Results

### The model and its dynamics

We consider two qubits undergoing decoherence via their interaction with a common harmonic oscillator environment. The dynamics of our two-qubit system can be described by the Hamiltonian $H = H_S + H_E + H_{SE}$ with (we take $\hbar = 1$ throughout)^67^

1 $$\begin{aligned} H_S&= \frac{\omega _0}{2}\left( \sigma _z ^{\left( 1\right) } +\sigma _z ^{\left( 2\right) }\right) ,\nonumber \\ H_E&= \sum _r \omega _r b_{r}^{\dagger } b_r, \nonumber \\ H_{SE}&=\left( \sigma _z ^{\left( 1\right) } + \sigma _z ^{\left( 2\right) }\right) \sum _{r} \left( g_{r}^{*}b_r + g_r b_r^\dagger \right) . \end{aligned}$$

Here $\sigma _z^{(1)}$ and $\sigma _z^{(2)}$ are the usual Pauli spin operators for the two qubits with $\omega _0$ the energy difference. $H_E$ is the harmonic oscillator environment Hamiltonian with the usual creation and annihilation operators (we have dropped the zero point energy for convenience), while $H_{SE}$ corresponds to the probe-environment interaction. Notice that any dissipative effects have been ignored; this can be justified because, generally, dissipation timescales are significantly longer than decoherence times^4,8,14^. Moreover, we have ignored any direct qubit-qubit interaction term; this is a common assumption made in studies of the effect of a common environment^14,68–72^. To obtain the dynamics of the two qubits, it is convenient to first transform our Hamiltonian to an interaction picture via the unitary operator $U_0(t)=e^{-i \left( H_E+H_S\right) t}$. We then obtain $H_{SE} \left( t\right) =\left( \sigma _z ^{\left( 1\right) } + \sigma _z ^{\left( 2\right) }\right) \sum _{r} \left( g_{r}^{*}b_r e^{-i\omega _r t} + g_r b_r^\dagger e^{i\omega _r t}\right)$. Using the Magnus expansion, this leads to the total unitary time-evolution operator (see the “Methods” section for the derivation)

2 $$\begin{aligned} U\left( t \right)&=\text {exp}\left\{ -i \left( \frac{\omega _0}{2}\left( \sigma _z ^{\left( 1\right) } + \sigma _z ^{\left( 2\right) }\right) + \sum _r \omega _r b_{r}^{\dagger } b_r \right) t\right\}\nonumber \\ &\times\text {exp}\Bigg \{\frac{1}{2}\left( \sigma _z ^{\left( 1\right) } + \sigma _z ^{\left( 2\right) }\right) \sum _r \left[ \alpha _r \left( t\right) b_r^\dagger - \alpha _r^* \left( t\right) b_r\right] -\frac{i}{2}\left( \mathbbm {1}+\sigma _z ^{\left( 1\right) } \sigma _z ^{\left( 2\right) }\right) \Delta \left( t \right) \Bigg \}, \end{aligned}$$

with $\alpha _r\left( t \right) =\frac{2g_r\left( 1-e^{i\omega _r t} \right) }{\omega _r}$, and $\Delta \left( t \right) =\sum _r \frac{4|{g_r}|^2}{\omega _r^2}\left[ \sin (\omega _r t) - \omega _r t\right]$. The reduced density operator of the two-qubit probe is obtained via $\rho _S \left( t\right) =\text {Tr}_E \left\{ U(t) \rho (0)U^\dagger (t) \right\}$. It is useful to express this reduced density operator in the eigenbasis of $\sigma _z ^{\left( 1\right) }$ and $\sigma _z ^{\left( 2\right) },$ that is, $|{k,l}\rangle$, where $\sigma _z ^{\left( 1\right) }|{k,l}\rangle = k |{k,l}\rangle$ and $\sigma _z ^{\left( 2\right) }|{k,l}\rangle = l |{k,l}\rangle$, with $k,l = \pm 1$. The matrix elements of the two-qubit density matrix are then

3 $$\begin{aligned} \left[ \rho _S \left( t\right) \right] _{k',l';k,l} =e^{-i\frac{\omega _0}{2} \left( k'+l'-k-l\right) t} e^{-i\frac{\Delta \left( t\right) }{2} \left( k'l'-kl\right) } \text {Tr}_{\textrm{S},\textrm{E}} \left\{ \rho \left( 0\right) e^{-R_{kl,k'l'}\left( t\right) }P_{kl,k'l'} \right\} , \end{aligned}$$

with $P_{kl,k'l'} \equiv |{k,l}\rangle \langle {k',l'}|, R_{kl,k'l'}\left( t\right) =\sum _r \left[ \widetilde{\alpha }_r\left( t\right) b_r^\dagger -\widetilde{\alpha }^*_r \left( t\right) b_r \right]$, and $\widetilde{\alpha }_r\left( t\right) =\frac{1}{2}\left( k+l-k'-l'\right) \alpha _r\left( t\right)$. To make further progress, we now assume that the total state is a simple product state with the two-qubit state being $\rho _S(0)$ and the environment in the thermal equilibrium state $\rho _E = \frac{e^{-\beta H_E}}{Z_E} \left( \text {here the partition function is } Z_E = \text {Tr}_E \left\{ e^{-\beta H_E} \right\} \right)$. That is, $\rho \left( 0\right) = \rho _S \left( 0\right) \otimes \rho _E$. The final state of the two-qubit probe is then found to be (see the “Methods” section)

$$\begin{aligned} \left[ \rho _S \left( t\right) \right] _{k',l';k,l} =\left[ \rho _S \left( 0\right) \right] _{k',l';k,l} e^{-i\frac{\omega _0}{2} \left( k'+l'-k-l\right) t} e^{-i\frac{\Delta \left( t\right) }{2} \left( k'l'-kl\right) } e^{ -\frac{1}{4}\left( k+l-k'-l'\right) ^2\Gamma \left( t\right) }. \end{aligned}$$

Note that here $\Gamma \left( t\right) = \sum _r \frac{4|g_r|^2}{\omega _r^2} [1 - \cos (\omega _r t)] \coth \left( \frac{\beta \omega _r}{2}\right)$ describes decoherence. In contrast, $\Delta (t)$ describes the indirect interaction between the qubits due to the interaction with the common environment. Since we are considering only decoherence, it is natural to take the initial state to be ‘pointing up’ along the *x*-axis, that is, $\rho _S\left( 0\right) =|{+,+}\rangle \langle {+,+}|,$ where $\sigma _x|{+}\rangle =|{+}\rangle$. Also, the effect of the environment on the system can be encapsulated by the spectral density of environment $J(\omega )$. This function effectively converts a sum over the environment modes to an integral via $\sum _r 4 |{g_r}|^2 f\left( \omega _r\right) \rightarrow \int _0^\infty d\omega \, J\left( \omega \right) f\left( \omega \right)$. Here, we consider the spectral density to be of the form $J\left( \omega \right) = G\frac{\omega ^s}{\omega _c^{s-1}}e^{-\frac{\omega }{\omega _c}}$, where *G* is the coupling strength, $\omega _c$ is the cutoff frequency, and *s* is the Ohmicity parameter with $s<1$, $s=1$ and $s>1$ representing sub-Ohmic, Ohmic, and super-Ohmic spectral densities respectively^3^. Finally, by taking a partial trace over the second qubit, the state of the first qubit alone is obtained as

4 $$\begin{aligned} \rho _{\textrm{S1}}^{\textrm{un}}\left( t\right)&= \begin{pmatrix} 1/2 &{} \frac{e^{-i\omega _0 t - \Gamma _{\textrm{un}}\left( t\right) }\cos \left[ \Delta \left( t\right) \right] }{2}\\ \frac{e^{i\omega _0 t - \Gamma _{\textrm{un}}\left( t\right) }\cos \left[ \Delta \left( t\right) \right] }{2} &{} 1/2 \end{pmatrix}, \end{aligned}$$

with $\Gamma _{\textrm{un}}\left( t\right) =\int _0^\infty \frac{J(\omega )}{\omega ^2} \left[ 1-\cos \left( \omega t \right) \right] \coth {\left( \frac{\beta \omega }{2}\right) d\omega }$ and $\Delta \left( t\right) =\int _0^\infty \frac{J(\omega )}{\omega ^2} \left[ \sin \left( \omega t \right) -\omega t\right] d\omega$. Note that we have added the subscript ‘un’ to emphasize that this is the decoherence factor when we have an uncorrelated initial state. Now, it is useful to split $\Gamma _{\textrm{un}}(t)$ into temperature-dependent and temperature-independent parts, that is, $\Gamma _{\textrm{un}}(t) = \Gamma _{\textrm{vac}}(t) + \Gamma _{\textrm{th}}(t)$. At zero temperature, $\Gamma _{\textrm{th}}(t) = 0$. On the other hand

5 $$\begin{aligned} \Gamma _{\textrm{vac}}(t)&={\left\{ \begin{array}{ll} \frac{G}{2}\ln \left( 1+ \omega ^2_c t^2\right) &{} s=1, \\ G \bar{\Gamma }[s-1] - \frac{1}{2}\left( \frac{G \bar{\Gamma }[s-1]}{\left( 1 - i\omega _c t\right) ^{s-1}} + \frac{G \bar{\Gamma }[s-1]}{\left( 1+i\omega _c t\right) ^{s-1}}\right) &{} s\ne 1, \end{array}\right. } \end{aligned}$$

where $\bar{\Gamma }$ is the usual gamma function defined as $\bar{\Gamma }[z]=\int _{0}^{\infty } t^{z-1} e^{-t} dt$.

We now consider taking into account the effect of the initial system-environment correlations. We assume the two qubits and their common environment have achieved a joint thermal equilibrium state. After that, at time $t = 0$, we perform a projective measurement on the system only to prepare the desired initial state of the probe $|{\psi }\rangle$. The total system-environment initial state is then written as

6 $$\begin{aligned} \rho \left( 0\right) =|{\psi }\rangle \langle {\psi }| \otimes \frac{\langle {\psi }| e^{-\beta H} |{\psi }\rangle }{Z}, \end{aligned}$$

where $Z = \text {Tr}_{\textrm{S},\textrm{E}}\left\{ e^{-\beta H} \right\}$ is the total partition function. The two-qubit probe’s subsequent dynamics are discussed in detail in the “Methods” section. Having worked these out, we once again obtain the state of the first qubit only by taking a partial trace over the second qubit. We write the final result as

7 $$\begin{aligned} \rho _{\textrm{S1}}^{\textrm{corr}} \left( t\right)&=\begin{pmatrix} 1/2 &{} \frac{e^{-i\xi \left( t\right) -\Gamma \left( t\right) }\cos \left[ \Delta \left( t\right) \right] }{2} \\ \frac{e^{i\xi \left( t\right) -\Gamma \left( t\right) }\cos \left[ \Delta \left( t\right) \right] }{2} &{} 1/2 \end{pmatrix}, \end{aligned}$$

where $\xi \left( t\right) =\omega _0 t + \chi \left( t\right)$. Again, $\Gamma \left( t\right)$ incorporates the decoherence effect of the environment, while $\Delta (t)$ captures the indirect interaction. Moreover, the effect of the initial correlations emerges as an effective level shift $\chi \left( t\right)$ as well as a modification of the decoherence factor. In particular, the decoherence factor is now $\Gamma \left( t\right) = \Gamma _{\textrm{un}}\left( t\right) +\Gamma _{\textrm{corr}}\left( t\right) ,$ with

8 $$\begin{aligned} \Gamma _{\textrm{corr}}\left( t\right) =\text {ln} \left[ \frac{1+e^{\beta \mathscr {C}}\cosh \left( \beta \omega _0\right) }{\sqrt{a^2 \left( t\right) + b^2 \left( t\right) }}\right] , \end{aligned}$$

while the effective level shift is $\chi \left( t\right) =\text {tan}^{-1} \left[ \frac{b \left( t\right) }{a \left( t\right) }\right]$. Here we have defined the time-dependent coefficients $a \left( t\right) = 1+e^{\beta \mathscr {C}}\cosh \left( \beta \omega _0\right) \cos [2\phi \left( t\right) ]$ and $b \left( t\right) = e^{\beta \mathscr {C}}\sinh \left( \beta \omega _0\right) \sin [2\phi (t)]$, with

9 $$\begin{aligned} \phi (t)= {\left\{ \begin{array}{ll} G\, \text {tan}^{-1}\left( \omega _c t\right) &{} \quad s=1,\\ \frac{G}{2i} \left( \frac{1}{\left( 1 - i\omega _c t\right) ^{s-1}} - \frac{1}{\left( 1+i\omega _c t\right) ^{s-1}}\right) \bar{\Gamma }[s-1] &{} \quad s\ne 1, \end{array}\right. } \end{aligned}$$

and $\mathscr {C} = \sum _r \frac{4|{g_r}|^2}{\omega _r}$. Note that we can get back the state in Eq. (4) if we set $\chi (t) = \Gamma _{\textrm{corr}}(t) = 0$ in Eq. (7).

### The quantum Fisher information

With the dynamics at hand, both with and without the initial correlations, we now move to calculate the quantum Fisher information (QFI). The QFI quantifies the precision with which a general environment parameter $\textit{x}$ can be estimated^73^. It can be shown that the QFI is related to the Cramer–Rao bound—the greater the QFI, the greater our precision of the estimate. The general expression for the QFI is given by

10 $$\begin{aligned} \mathbb {F}_{Q}\left( x\right) =\sum _{n=1}^2 \frac{(\partial _x\rho _n)^2}{\rho _n}+2\sum _{n\ne m }\frac{(\rho _n-\rho _m)^2}{\rho _n+\rho _m} |{\langle {\varepsilon _m}|{\partial _x\varepsilon _n}\rangle }|^2, \end{aligned}$$

where $|{\rho _n}\rangle$ is the $n^{\textrm{th}}$ eigenstate of our reduced single-qubit state and $\rho _n$ is the corresponding eigenvalue. For the evaluated single qubit $2 \times 2$ matrix, it is straightforward to calculate the eigenvalues and eigenstates. We find that $\rho _1=\frac{1}{2}[1-\mathcal {F}\left( t\right) ]$ and $\rho _2=\frac{1}{2}[1+\mathcal {F}\left( t\right) ]$ with $\mathcal {F}\left( t\right) = \cos \left[ \Delta (t)\right] e^{-\Gamma \left( t\right) }$. The corresponding eigenstates are

11 $$\begin{aligned} |{\rho _1(t)}\rangle&=\frac{1}{\sqrt{2}}\left\{ |{+1}\rangle + e^{i\xi \left( t\right) }|{-1}\rangle \right\} , \nonumber \\ |{\rho _2(t)}\rangle&=\frac{1}{\sqrt{2}}\left\{ |{+1}\rangle - e^{i\xi \left( t\right) }|{-1}\rangle \right\} , \end{aligned}$$

where $|{+1}\rangle$ and $|{-1}\rangle$ being the eigenstates of $\sigma _z$. Now $\left( \partial _x \rho _1 \right) ^2 =\left( \partial _x \rho _2 \right) ^2 =\frac{1}{4}e^{-2\Gamma } \left( \sin \Delta \partial _x \Delta + \cos \Delta \partial _x \Gamma \right) ^{2}.$ Calculating also the derivatives of the eigenstates and substituting in Eq. (10), the QFI comes out to be

12 $$\begin{aligned} \mathbb {F}_{Q}\left( x\right) =\frac{\left( \sin {\Delta } \partial _x \Delta + \cos {\Delta } \partial _x\Gamma \right) ^2}{e^{2\Gamma }-\cos ^2{\Delta }} + \frac{\cos ^2{\Delta } \left( \partial _x \chi \right) ^2}{e^{2\Gamma }}. \end{aligned}$$

This expression gives the QFI for our two-qubit scheme where we take the initial correlations into account, and it reduces to the expression for a single-qubit case by setting $\Delta = 0$^66^. If we start with the simple product state without taking the initial correlations into account, then we can obtain the QFI by setting $\chi = 0$ and replacing $\Gamma$ by $\Gamma _{\textrm{un}}$ in Eq. (12). Note also that the presence of a direct qubit-qubit interaction term of the form $\frac{\kappa }{2}\sigma _z^{(1)}\sigma _z^{(2)}$ simply leads to $\Delta (t)$ being replaced by $\Delta (t) + \kappa$ in Eq. (12). Our results should then not change appreciably unless $\kappa$ becomes comparable to the indirect interaction. Before moving on to concrete examples of estimating the environment parameters, it is already clear that the addition of the indirect qubit-qubit interaction appears highly promising in increasing the QFI.

### Estimation of the cutoff frequency of the environment

As the first example of applying our expression of the QFI [see Eq. (12)], we now look in detail at estimating the environment’s cutoff frequency $\omega _c$. We first note that (at zero temperature)

**Figure 1:**
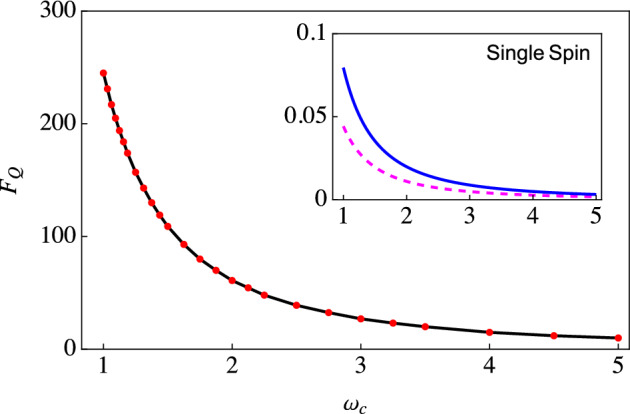
The main figure shows the behavior of the optimized QFI using our two-qubit scheme for estimating the cutoff frequency as a function of the cutoff frequency. The black, solid curve is obtained by including the effects of the initial correlations, while the dotted, red curve ignores these effects. We have taken $\omega _0 = 1$ and the rest of the parameters are $G= 0.01$, $s=0.5$, and the temperature $T = 0$. The inset shows the optimized QFI if we use a single qubit or spin (without any second qubit), both with (solid, blue curve) and without initial correlations (dashed, magenta curve). The parameters used are the same as the main figure.

**Figure 2:**
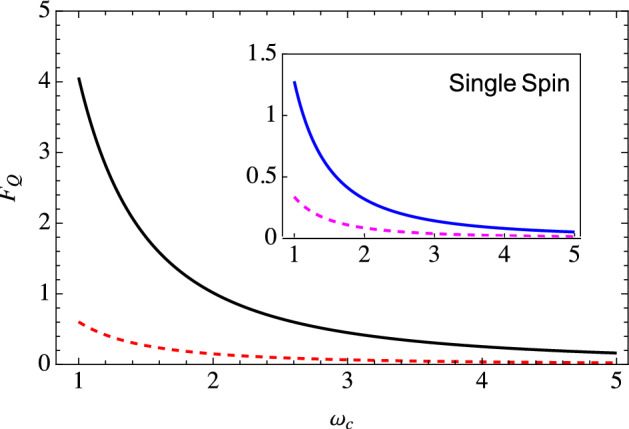
Similar to Fig. 1, the main figure shows the behavior of the optimized quantum Fisher information for estimating the cutoff frequency with our two-qubit probe scheme. In contrast, the inset shows the optimized quantum Fisher information if we used only a single-qubit probe. Here $G = 1$, while the rest of the parameters are the same as Fig. 1.

**Figure 3:**
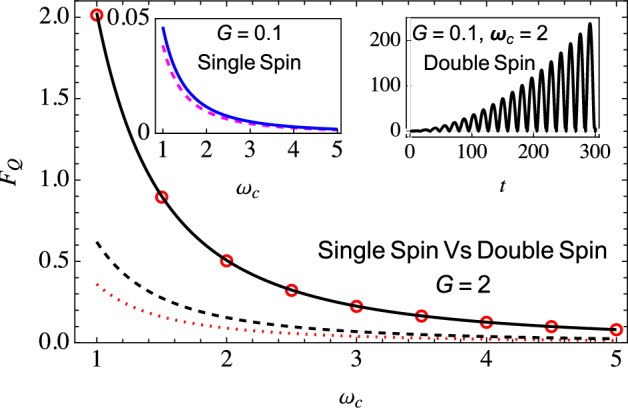
Comparison of the optimized QFI for estimating $\omega _c$ for a single-qubit probe versus our two-qubit scheme for an Ohmic environment $(s=1)$. In the main plot, the solid black (with initial correlations) and dashed black (without initial correlations) curves show the optimized QFI for the two-qubit scheme. In contrast, the red circles (with initial correlations) and dotted red curve (without initial correlations) show the optimized QFI for the single-qubit probe. In the top-left inset, the optimized QFI is plotted with (blue solid curve) and without (dashed magenta curve) initial correlations for the single-qubit probe ($G=0.1$). In contrast, in the top-right inset, the QFI is plotted with (black solid curve) and without (dashed red curve) initial correlations ($G=0.1$ and $\omega _c = 2$) for the two-qubit scheme (these curves overlap). A qualitatively similar increase in Fisher information is observed with $G = 0.1$ for other values of the cutoff frequency $\omega _c$. Other parameters are the same as Fig. 1.

**Figure 4:**
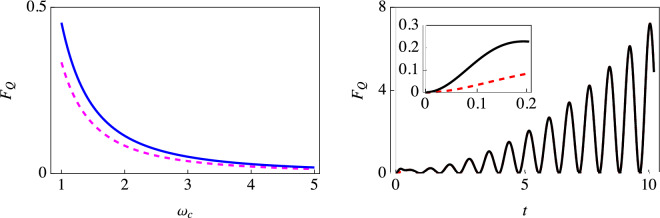
Behavior of the optimized QFI with a super-Ohmic ($s = 2$) environment. The left plot shows the optimized QFI for estimating $\omega _c$ with (solid blue curve) and without (dashed magenta curve) initial correlations using a single-qubit probe. The right plot shows the optimized QFI using our two-qubit scheme, with (solid black curve) and without (dashed red curve) initial correlations (here $\omega _c = 2$). The inset zooms in on the behavior of the optimized QFI for small values of time. Similar qualitative behavior is observed for other values of $\omega _c$. We have set $\omega _0 = 1$, with the environment coupling strength $G = 2$ and temperature $T = 0$.

13 $$\begin{aligned} \frac{\partial \Gamma }{\partial \omega _c}&={\left\{ \begin{array}{ll} \frac{G \omega _c t^2}{1 + \omega ^{2}_c t^2} &{} \quad\quad s = 1, \\ iG \bar{\Gamma }[s]t\left( \frac{1}{2\left( 1+i\omega _c t\right) ^s} - \frac{1}{2\left( 1-i\omega _c t\right) ^s}\right) &{} \quad\quad s \ne 1, \end{array}\right. } \nonumber \\ \frac{\partial \Delta }{\partial \omega _c}&={\left\{ \begin{array}{ll} \frac{G \omega _c^2 t^3}{1 + \omega ^{2}_c t^2} &{} \quad s = 1, \\ G \bar{\Gamma }[s]t\left( \frac{1/2}{\left( 1+i\omega _c t\right) ^s} + \frac{1/2}{\left( 1-i\omega _c t\right) ^s} - 1 \right) &{} \quad s \ne 1, \end{array}\right. } \nonumber \\ \frac{\partial \chi }{\partial \omega _c}&={\left\{ \begin{array}{ll} \frac{2G t}{1 + \omega ^{2}_c t^2} &{} \quad\quad s = 1, \\ -G \bar{\Gamma }[s]t\left( \frac{1}{\left( 1+i\omega _c t\right) ^s} + \frac{1}{\left( 1-i\omega _c t\right) ^s}\right) &{} \quad\quad s \ne 1. \end{array}\right. } \end{aligned}$$

Using these in Eq. (12), we obtain the quantum Fisher information to estimate the cutoff frequency as a function of time. We then optimize this QFI over the interaction time to find the maximum possible QFI. For example, one could plot the QFI as a function of time for different values of $\omega _c$ and thereby note the maximum value of QFI for each value of $\omega _c$. We can then investigate the behavior of this optimal QFI as a function of the cutoff frequency. This behavior is illustrated in Fig. 1. The main figure shows the typical behavior of the QFI for estimating the cutoff frequency for a sub-Ohmic environment using our two-qubit scheme, both with and without including the effect of the initial correlations. It is clear that in this weak coupling strength regime, the effect of the initial correlations is insignificant, as expected since the black, solid curve overlaps with the red dotted curve. The inset shows the optimized QFI if we use a single qubit interacting with the environment with the same set of parameters. What is most notable is the drastic increase of the QFI with our two-qubit scheme compared to using a single qubit—it is a three orders of magnitude increase, demonstrating the advantage of using our two-qubit scheme remarkably. This is far beyond what one would naively expect when using a two-qubit probe as compared to a single-qubit probe. Moreover, we are still performing measurements on a single qubit and computing the QFI via a single-qubit density matrix. The increase is simply because of the indirect qubit-qubit interaction (the $\Delta$ term); this term is, of course, completely absent with a single-qubit probe. Interestingly, if we increase the coupling strength *G*, our two-qubit scheme improves the QFI, although the increase is not as drastic as in the case of weak coupling (see Fig. 2)—the increased decoherence leads to smaller values of the QFI.

We also investigated an Ohmic environment (see Fig. 3). For strong coupling, we notice the overlap of red circles (using the simple single-qubit probe with correlations included) and the solid black curve (using our two-qubit scheme with the effect of the correlations included), thereby indicating that the two schemes perform similarly for strong coupling with an Ohmic environment. However, the situation drastically changes for weaker coupling. As one can see from the inset, very surprisingly, the QFI with our two-qubit scheme keeps increasing as the qubits interact with their environment. The decoherence is now smaller, and the indirect interaction leads to a buildup of the information gained about the environment. This is in complete contrast with the QFI obtained with a single-qubit probe, which is bounded (see the left inset). The continuous increase in the QFI is also simple to explain mathematically. From the definitions of $\Gamma (t)$ and $\Delta (t)$, and the expressions given in Eq. (13), it is easy to see that for the Ohmic environment at long times, $\frac{\partial \chi }{\partial \omega _c} \rightarrow 0$, $\frac{\partial \Gamma }{\partial \omega _c} \rightarrow \frac{G}{\omega _c}$, $\frac{\partial \Delta }{\partial \omega _c} \rightarrow Gt$, $\sin \Delta \rightarrow \sin [G(\frac{\pi }{2} - \omega _c t)]$, and $e^{2\Gamma } \rightarrow (\omega _c t)^{2G}$. It is then clear that at long times, using Eq. (12), the temporal dependence of the quantum Fisher information is captured by $\sin ^2[G(\frac{\pi }{2} - \omega _c t)] t^{2(1 - G)}$. This means that the quantum Fisher information for the Ohmic environment will keep on increasing for $G < 1$, while it will not for $G > 1$. This is precisely in agreement with what we have observed in Fig. 3. With a super-Ohmic environment (see Fig. 4), the indirect inter-qubit interaction (the $\Delta$ term) again plays a vital role in leading to a continuous increase in the QFI. Moreover, with the super-Ohmic environment, the buildup of QFI with the two-qubit scheme can persist even in the strong coupling regime.

### Estimation of the system-environment coupling strength

We now consider estimating the coupling strength *G*. Again, we use the expression in Eq. (12) and optimize it over the interaction time to get optimized QFI. We now need the derivatives (evaluated at zero temperature)

14 $$\begin{aligned} \frac{\partial \Gamma }{\partial G}&={\left\{ \begin{array}{ll} \frac{1}{2}\ln \left( 1 + \omega ^{2}_c t^2\right) &{} \quad s = 1, \\ \bar{\Gamma }[s-1] - \left( \frac{\bar{\Gamma }[s-1]/2}{\left( 1 - i\omega _c t\right) ^{s-1}} + \frac{\bar{\Gamma }[s-1]/2}{\left( 1 + i\omega _c t\right) ^{s-1}}\right) &{} \quad s \ne 1, \end{array}\right. } \nonumber \\ \frac{\partial \Delta }{\partial G}&={\left\{ \begin{array}{ll} \text {tan}^{-1}\left( \omega _c t\right) - \omega _c t &{} \quad s=1, \\ \bar{\Gamma }[s] \omega _c t - \left( \frac{i\bar{\Gamma }[s-1]/2}{\left( 1-i\omega _c t\right) ^{s-1}} - \frac{i\bar{\Gamma }[s-1]/2}{\left( 1 + i\omega _c t\right) ^{s-1}} \right) &{} \quad s\ne 1, \end{array}\right. }\nonumber \\ \frac{\partial \chi }{\partial G}&={\left\{ \begin{array}{ll} 2\text {tan}^{-1}\left( \omega _c t\right) &{} \quad s=1, \\ -i\bar{\Gamma }[s-1] \left( \frac{1}{\left( 1 - i\omega _c t\right) ^{s-1}} - \frac{1}{\left( 1+i\omega _c t\right) ^{s-1}}\right) &{} \quad s \ne 1. \end{array}\right. } \end{aligned}$$

**Figure 5:**
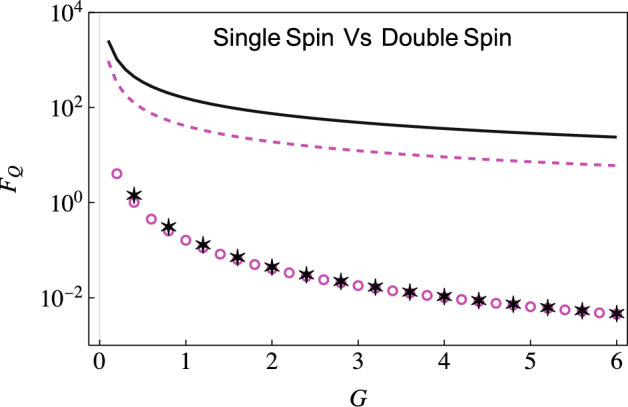
Behavior of optimized QFI versus coupling strength *G* obtained using a single qubit (or spin) probe with (dashed, magenta curve) and without (magenta circles) initial correlations, as well as with our two-qubit scheme (the solid, black curve is with initial correlations, while the black asterisks show the QFI without initial correlations). Here, we have considered a sub-Ohmic ($s=0.1$) environment. We have set $\omega _0 = 1$, and $\omega _{c}=5$ and temperature $T = 0$.

**Figure 6:**
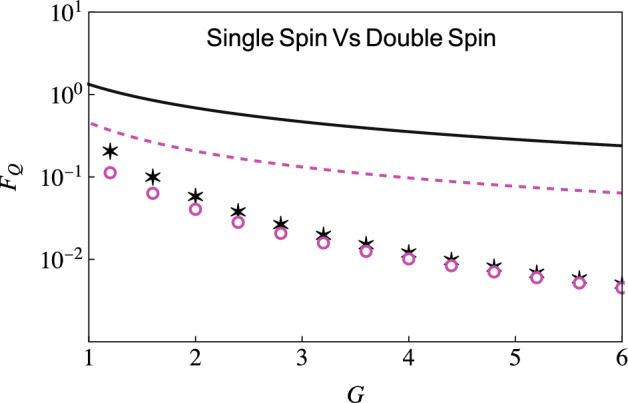
Similar to Fig. 5, we plot the behavior of the optimized QFI versus the coupling strength *G*. The solid black curve shows the optimized QFI with our two-qubit probe scheme if we include the effect of the initial correlations; the black asterisks illustrate the QFI with the two-qubit probe scheme but without including the effect of the initial correlations. We also show the optimized QFI using a single-qubit probe with (dashed magenta curve) and without (the magenta circles) including the effect of the initial correlations. Here we consider an Ohmic environment ($s=1$). The rest of the parameters are the same as Fig. 5.

**Figure 7:**
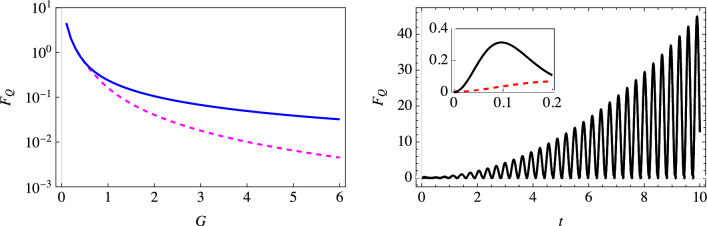
Behavior of the optimized QFI for the estimation of the coupling strength *G* with a super-Ohmic ($s = 2$) environment. The left plot shows the optimized QFI for estimating *G* with (solid blue curve) and without (dashed magenta curve) initial correlations using a single-qubit probe. The right plot shows the optimized QFI using our two-qubit scheme, with (solid black curve) and without (dashed red curve) initial correlations (here $G = 2$). The inset zooms in on the behavior of the optimized QFI for small values of time. We have set $\omega _0 = 1$, with the cutoff frequency $\omega _c = 5$ and temperature $T = 0$.

We first compare the optimized QFI for estimating the coupling strength *G* obtained using our two-qubit scheme with the QFI obtained using a single-qubit probe for a sub-Ohmic environment. Results are illustrated in Fig. 5, where we have shown the behavior of the optimized QFI versus the coupling strength *G* using a single-qubit probe both with and without incorporating the effect of the initial correlations—these are shown with the dashed, magenta curve and the circular markers respectively. We have also shown the QFI with our two-qubit scheme, both with (solid, black curve) and without (the asterisk markers) including the initial correlations. At least three points should be noted here. First, if we ignore the initial correlations, there is little difference between the two schemes. Second, the role of the initial correlation is, in general, vital. Third, with both indirect interaction and the initial correlations accounted for, there is a drastic increase in the QFI compared to the use of a single-qubit probe. Following the same color scheme and parameters used in Fig. 5, we demonstrate the optimized QFI in an Ohmic environment $s=1$ as well (see Fig. 6). With this environment, while the QFI is lower than the sub-Ohmic environment, the benefit of using our two-qubit scheme is still evident. The advantage of our two-qubit scheme becomes even more evident with super-Ohmic environments (see Fig. 7). Once again, the QFI generally keeps increasing as we increase the interaction time (see the right figure in Fig. 7) for our two-qubit scheme. If we compare this with the results obtained using a single-qubit probe with (solid blue curve) or without (magenta dashed curve) correlations (see the left plot), we see that the QFI for the single-qubit probe is far smaller.

### Estimation of temperature

**Figure 8:**
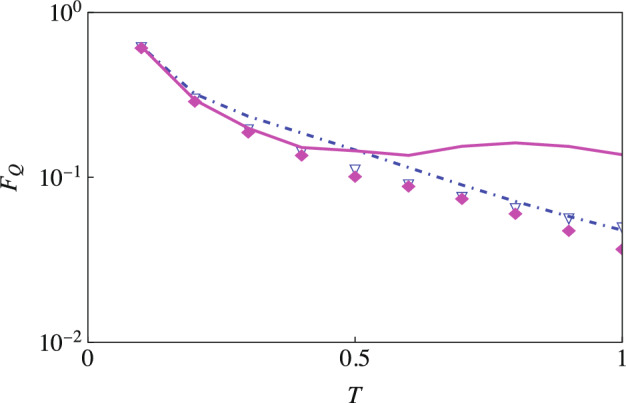
The optimized QFI for estimating temperature. The solid magenta curve (with initial correlations) and the magenta diamond markers (without initial correlations) denote the optimized QFI with an Ohmic environment $(s = 1)$ and our two-qubit scheme. The dot-dashed blue curve (with initial correlations) and blue triangle markers (without initial correlations) show the optimized QFI with a single qubit probe. Here we have $\omega _0 = 1$, $\omega _c = 5$ and $G = 0.5$.

We now consider the estimation of temperature using a single-qubit probe and our two-qubit scheme. Since temperature is not zero here, $\Gamma _{\textrm{corr}}\left( t\right)$ and $\Gamma _{\textrm{th}}(t)$ are no longer zero. $\Gamma _{\textrm{corr}}\left( t\right)$ can be found analytically—its expression is given in Eq. (8)—while $\Gamma _{\textrm{th}}(t)$ and its temperature derivative are found numerically. We illustrate our results in Fig. 8 for an Ohmic environment. The key point to note here is that now we do not observe as much of an increase in the Fisher information via our two-qubit scheme as we had observed previously. This is simply because the indirect interaction $\Delta (t)$ is independent of temperature; as such, we expect the Fisher information for the estimation of temperature to not change very significantly if we use our two-qubit scheme rather than a single qubit probe. We have checked that this is the case with sub-Ohmic and super-Ohmic environments as well.

**Figure 9:**
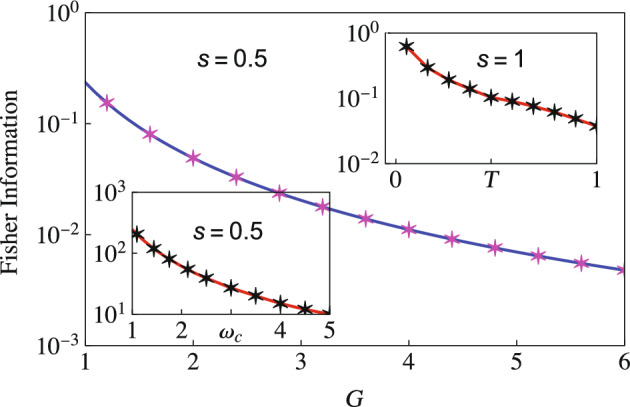
Plot of QFI (solid curves) versus the classical Fisher information (asterisk markers). The main plot shows the estimation of the coupling strength *G* with $\omega _c=5$ and temperature $T = 0$. We have again plotted the optimized Fisher information (quantum and classical) in the insets. On the top right, we estimate the temperature *T* with coupling strength $G=0.5$ and cutoff frequency $\omega _c = 5$. At the bottom left, we estimate the cutoff frequency with coupling strength $G=0.01$ and temperature $T = 0$.

### Optimal measurement

Until now, we have found that the QFI can be drastically increased by using our two-qubit scheme. The question remains regarding which measurements need to be performed to obtain this maximum QFI. This can be answered by calculating the classical Fisher information (CFI) for a particular measurement scheme; if the CFI comes out to be equal to the QFI, then we have found the optimal measurement to be performed. We guess that the optimal measurements are projective measurements described by the projection operators $\textbf{P}_1 =|{\Psi _1}\rangle \langle {\Psi _1}|$ and $\textbf{P}_2 =|{\Psi _2}\rangle \langle {\Psi _2}|$, with

15 $$\begin{aligned} |{\Psi _1}\rangle&=\frac{1}{\sqrt{2}} \left\{ |{+1}\rangle + e^{i\varphi }|{-1}\rangle \right\} , \nonumber \\ |{\Psi _2}\rangle&=\frac{1}{\sqrt{2}} \left\{ |{+1}\rangle - e^{i\varphi }|{-1}\rangle \right\} . \end{aligned}$$

Here, $\varphi$ is the usual angle in the Bloch sphere representation. The effect of this measurement is encapsulated by the conditional probability $\mathbb {P}(k|x)$ of getting measurement result *k* with $k = 1, 2$ (corresponding to the post-measurement states $|{\Psi _1}\rangle$ and $|{\Psi _2}\rangle$), and *x* is the parameter we intend to estimate. For the discrete case, the classical Fisher information (CFI) is^74^

16 $$\begin{aligned} \mathbb {F}_{c}(x) = \sum ^{2}_{k=1} \left( \partial ^{2}_{x} \ln \left[ \mathbb {P}(k|x)\right] \right) \mathbb {P}(k|x), \end{aligned}$$

where $\partial ^{2}_{x}$ denotes the double derivative with respect to the parameter *x* that is to be estimated. These probabilities are calculated via the usual Born rule. Using the projection operators along with the final state [see Eq. (7)], we find that (we have set $\Theta = \chi +\omega _0 t -\varphi$ to show a more compact form)

17 $$\begin{aligned} \mathbb {F}_{c}(x) =\frac{\left( \left( \frac{\partial {\Delta }}{\partial x} \sin \Delta + \frac{\partial {\Gamma }}{\partial x} \cos \Delta \right) \cos \Theta - \frac{\partial {\chi }}{\partial x}\cos \Delta \sin \Theta \right) ^{2}}{e^{2\Gamma } - \cos ^{2}\Delta \cos ^{2}\Theta }. \end{aligned}$$

If we disregard the effect of the initial correlations, then this expression reduces to

18 $$\begin{aligned} \mathbb {F}^{\textrm{woc}}_{c}(x) =\frac{\left( \frac{\partial {\Delta }}{\partial x} \sin \Delta + \frac{\partial {\Gamma }}{\partial x} \cos \Delta \right) ^{2}}{\sec ^{2}\left( \omega _0 t -\varphi \right) e^{2\Gamma } - \cos ^{2}\Delta }. \end{aligned}$$

We aim to maximize the classical Fisher information [see Eq. (17)] over the angle $\varphi$. If the effect of initial correlations is not included, then it is clear that $\varphi =\omega _0 t$ is the optimal value. In this case, the CFI reduces to the QFI, so we have found the optimal measurement. On the other hand, if $\chi \ne 0$, we can show that for

19 $$\begin{aligned} \varphi = \omega _0 t + \chi - \text {tan}^{-1} \left[ \frac{\partial _{x}{\chi } \cos \Delta \left( e^{2\Gamma } - \cos ^{2} \Delta \right) }{e^{2\Gamma } \left( \partial _{x}{\Delta } \sin \Delta + \partial _{x}{\Gamma } \cos \Delta \right) } \right] , \end{aligned}$$

the CFI reduces to the QFI. Again, this means that we have managed to find the optimal measurement. We further support these claims by plotting both the QFI and CFI while estimating the environment’s cutoff frequency $\omega _c$, system-environment coupling strength *G* and the environment’s temperature *T* (see Fig. 9), where the overlap between the CFI and the QFI shows that we have successfully found the optimal measurements to be performed.

## Discussion

In conclusion, we have explored the use of a two-qubit probe scheme to estimate the parameters of a harmonic oscillator environment. The key idea is that both qubits of the probe interact with the common environment. This common environment induces an indirect interaction between the qubits. The dynamics of only one of these qubits then depend sensitively on this indirect interaction on top of the decoherence caused by the environment and the effect of the initial system-environment correlations. Consequently, we can expect our two-qubit scheme to estimate the environment parameters better than simply using a single-qubit probe. We illustrated that this is indeed the case by evaluating the optimal quantum Fisher information. The increase in the quantum Fisher information can be unexpectedly dramatic. Our work conclusively shows that two-qubit probes can indeed be far more advantageous in estimating the environment parameters than a single-qubit probe.

## Methods

### Derivation of unitary time-evolution operator

The total unitary time-evolution operator for the two-qubit probe can be written as $U(t) = e^{-iH_S t} e^{-i H_E t} U_{SE}(t)$. To find the unitary time evolution operator $U_{SE}(t)$ corresponding to $H_{SE}(t)$, we use the Magnus expansion $U_{SE}\left( t\right) = \text {exp}\left\{ \sum _{i=1}^\infty A_i\left( t\right) \right\} .$ The integrals $A_1, A_2, \ldots$ are evaluated below. For simplicity, we let $\widetilde{J}_z=\sigma _z ^{\left( 1\right) } + \sigma _z ^{\left( 2\right) }$. We first have

20 $$\begin{aligned} A_1&=-i \int _0^t H_{SE} \left( t_1\right) dt_1, \nonumber \\&=-i \widetilde{J}_z \sum _r \int _0^t \left( g_{r}^{*}b_r e^{-i\omega _r t} + g_r b_r^\dagger e^{i\omega _r t}\right) dt_1, \nonumber \\&=\frac{\widetilde{J}_z}{2} \sum _r\left[ \alpha _r \left( t\right) b_r^\dagger - \alpha _r^* \left( t\right) b_r\right] , \end{aligned}$$

with $\alpha _r\left( t \right) =\frac{2g_r}{\omega _r}\left( 1-e^{i\omega _r t} \right) .$ Now in order to calculate $A_2$, we first determine the commutator $\left[ H_{SE} \left( t_1\right) ,H_{SE} \left( t_2\right) \right]$ which comes out to be $4i\left( \mathbbm {1}+\sigma _z ^{\left( 1\right) } \sigma _z ^{\left( 2\right) }\right) \sum _r |{g_r}|^2 \sin \left[ \omega _r (t_2-t_1)\right] .$ Therefore

21 $$\begin{aligned} A_2&=-\frac{1}{2} \int _0^t dt_1\int _0^{t_1} \left[ H_{SE} \left( t_1\right) ,H_{SE} \left( t_2\right) \right] \, dt_{2},\nonumber \\&= -\frac{i}{2}\left( \mathbbm {1}+\sigma _z ^{\left( 1\right) } \sigma _z ^{\left( 2\right) }\right) \Delta \left( t \right) , \end{aligned}$$

with $\Delta \left( t \right) =\sum _r \frac{4|{g_r}|^2}{\omega _r^2}\left[ \sin (\omega _r t) - \omega _r t\right] .$ This is simply a c-number, which means that $A_3=A_4=A_{5}= \cdots =0$. The expression of the exact unitary time-evolution operator can then be written as

$$\begin{aligned} U\left( t \right)&=\text {exp}\left\{ -i \left( \frac{\omega _0}{2} \left( \sigma _z ^{\left( 1\right) } + \sigma _z ^{\left( 2\right) }\right) + \sum _r \omega _r b_{r}^{\dagger } b_r \right) t\right\} \\& \times \text {exp}\Bigg \{\frac{1}{2}\left( \sigma _z ^{\left( 1\right) } + \sigma _z ^{\left( 2\right) }\right) \sum _r\left[ \alpha _r \left( t\right) b_r^\dagger - \alpha _r^* \left( t\right) b_r\right] -\frac{i}{2}\left( \mathbbm {1}+\sigma _z ^{\left( 1\right) } \sigma _z ^{\left( 2\right) }\right) \Delta \left( t \right) \Bigg \}. \end{aligned}$$

### Dynamics with factorized initial state

To make further progress, we now assume that the total state is a product state. In other words, we denote the initial state of the two qubits comprising the probe as $\rho _S(0)$ and the total state as $\rho (0) = \rho _S \left( 0\right) \otimes \rho _E$, where $\rho _E = \frac{e^{-\beta H_E}}{Z_E} \ \text {with} \ Z_E = \text {Tr}_E \left\{ e^{-\beta H_E} \right\} .$ From Eq. (3), we then have

22 $$\begin{aligned} \left[ \rho _S \left( t\right) \right] _{k',l';k,l} =\left[ \rho _S \left( 0\right) \right] _{k',l';k,l} \text {Tr}_{E} \left\{ \rho _Ee^{-R_{kl,k'l'} \left( t\right) }\right\} e^{-\frac{i \omega _0}{2} \left( k'+l'-k-l\right) t} e^{-\frac{i\Delta \left( t\right) }{2} \left( k'l'-kl\right) }. \end{aligned}$$

We now simplify $\text {Tr}_{E} \left\{ \rho _Ee^{-R_{kl,k'l'}\left( t\right) }\right\}$. Since the modes of the harmonic oscillator are independent of each other, we obtain

23 $$\begin{aligned} \left\langle {e^{-R_{kl,k'l'}\left( t\right) }}\right\rangle = \prod _r \text {exp} \left\{ -\frac{1}{2} |{\widetilde{\alpha }_r\left( t\right) }|^2 \left\langle {2n_r + 1 }\right\rangle \right\} , \end{aligned}$$

where we have defined $n_r = \left\langle {b_r^\dagger b_r}\right\rangle$. The environment being in thermal equilibrium, $n_r$ is simply the Bose-Einstein distribution, that is, $n_r =\frac{1}{e^{{\beta \omega _r }} -1}=\frac{1}{2}\left\{ \coth \left( \frac{\beta \omega _r}{2}\right) -1\right\}$. Therefore

$$\begin{aligned} \text {Tr}_{E} \left\{ \rho _Ee^{-R_{kl,k'l'}\left( t\right) }\right\} = \text {exp}\left\{ -\frac{1}{4}\left( k+l-k'-l'\right) ^2\Gamma \left( t\right) \right\} , \end{aligned}$$

with $\Gamma \left( t\right) =\sum _r \frac{ 4|{g_r}|^2}{\omega _r^2} \left[ 1-\cos \left( \omega _r t \right) \right] \coth {\left( \frac{\beta \omega _r}{2}\right) }.$ To sum up, the final state of the two-qubit probe can be written

24 $$\begin{aligned} \left[ \rho _S \left( t\right) \right] _{k',l';k,l} =\left[ \rho _S \left( 0\right) \right] _{k',l';k,l} e^{-i\frac{\omega _0}{2} \left( k'+l'-k-l\right) t} e^{-i\frac{\Delta \left( t\right) }{2} \left( k'l'-kl\right) } e^{ -\frac{1}{4}\left( k+l-k'-l'\right) ^2\Gamma \left( t\right) }. \end{aligned}$$

### Dynamics with correlated initial state

We now prepare the initial system-environment state with a projective measurement described by the projection operator $|{\psi }\rangle \langle {\psi }|$. The initial joint system-environment state can then be written as

25 $$\begin{aligned} \rho \left( 0\right) =|{\psi }\rangle \langle {\psi }| \otimes \frac{\langle {\psi }| e^{-\beta H} |{\psi }\rangle }{Z}, \end{aligned}$$

where $Z = \text {Tr}_{\textrm{S},\textrm{E}}\left\{ e^{-\beta H} \right\}$ is the total partition function. We label the joint eigenstates of $\sigma _z^{(1)}$ and $\sigma _z^{(2)}$ as $|{p,q}\rangle$ such that $\sigma ^{(1)}_z |{p,q}\rangle = p |{p,q}\rangle$ and $\sigma ^{(2)}_z |{p,q}\rangle = q |{p,q}\rangle$ with $p,q=\pm 1$. Inserting the completeness relation $\sum _{p,q} |{p,q}\rangle \langle {p,q}| = \mathbbm {1}$, we find the total partition function to be

$$\begin{aligned} Z&= \sum _{p,q}\text {Tr}_{\textrm{S},\textrm{E}} \Big \{ e^{-\frac{\beta \omega _0}{2}\left( \sigma _z ^{\left( 1\right) } + \sigma _z ^{\left( 2\right) }\right) } e^{-\beta \left\{ H_E+\left( \sigma _z ^{\left( 1\right) } + \sigma _z ^{\left( 2\right) }\right) \sum _{r} \left( g_{r}^{*}b_r + g_r b_r^\dagger \right) \right\} } |{p,q}\rangle \langle {p,q}| \Big \}, \\&=\sum _{p,q}\text {Tr}_{\textrm{S},\textrm{E}} \Big \{ e^{-\frac{\beta \omega _0}{2}\left( p+q\right) } e^{-\beta \left\{ H_E+\left( p+q\right) \sum _{r} \left( g_{r}^{*}b_r + g_r b_r^\dagger \right) \right\} } |{p,q}\rangle \langle {p,q}| \Big \}, \\&= \sum _{p,q} e^{-\frac{\beta \omega _0}{2}\left( p+q\right) } \text {Tr}_E\left\{ e^{-\beta \left\{ H_E+\left( p+q\right) \sum _{r} \left( g_{r}^{*}b_r + g_r b_r^\dagger \right) \right\} } \right\} = \sum _{p,q}e^{-\frac{\beta \omega _0}{2}\left( p+q\right) } \text {Tr}_E\left\{ e^{-\beta H_E^{\left( p,q\right) }} \right\} . \end{aligned}$$

Here we have defined the shifted Hamiltonian $H_E^{\left( p,q\right) } = H_E+\left( p+q\right) \sum _{r} \left( g_{r}^{*}b_r + g_r b_r^\dagger \right) .$ To further simplify $H_E^{\left( p,q\right) }$, we use displaced harmonic oscillator modes $B_{r,p,q} = b_r + \frac{\left( p+q\right) g_r}{\omega _r}.$ Now, the trace over the environment becomes $\text {Tr}_{E}\left\{ e^{-\beta H_E^{\left( p,q\right) }} \right\} =e^{\beta \sum _r(p+q)^2\frac{|{g_r}|^2}{\omega _r}}Z_E$, with $Z_E=\text {Tr}_{E}\left\{ e^{-\beta \sum _r \left\{ \omega _r B_{r,p,q}^\dagger B_{r,p,q} \right\} } \right\}$. Therefore, the final form of the partition function is

26 $$\begin{aligned} Z=\sum _{p,q}e^{-\frac{\beta \omega _0}{2}\left( p+q\right) } e^{\beta \sum _r(p+q)^2\frac{|{g_r}|^2}{\omega _r}}Z_E. \end{aligned}$$

Similarly, we can simplify the time-dependent factor $R_{kl,k'l'}\left( t\right)$ present in Eq. (3) in terms of the displaced harmonic oscillator modes. We get

$$\begin{aligned} R_{kl,k'l'}\left( t\right) =\sum _r \left[ \widetilde{\alpha }_r \left( t\right) B_{k,l}^\dagger -\widetilde{\alpha }^*_r \left( t\right) B_{k,l} \right] + i(p+q) \widetilde{\Phi } \left( t\right) , \end{aligned}$$

where $\widetilde{\Phi }\left( t\right) =\frac{1}{2}\left( k+l-k'-l'\right) \phi \left( t\right) ,$ with $\phi \left( t\right) = \sum _r \frac{4|{g_r}|^2}{\omega _r^2}\sin \left( \omega _r t\right) \rightarrow \int _0^\infty G\frac{\omega ^s}{\omega _c^{s-1}}e^{-\frac{\omega }{\omega _c}} \frac{\sin \left( \omega t \right) }{\omega ^2}d\omega$. To simplify Eq. (3), starting from the initial state in Eq. (6), we note that

$$\begin{aligned}&\text {Tr}_{\textrm{S},\textrm{E}} \left\{ \rho \left( 0\right) e^{-R_{kl,k'l'}\left( t\right) }P_{kl,k'l'} \right\} \\&\quad =\frac{1}{Z}\sum _{p,q}e^{-\frac{\beta \omega _0}{2}\left( p+q\right) } Z_E \langle {p,q}|{\psi }\rangle \langle {\psi }| P_{kl,k'l'} |{\psi }\rangle \langle {\psi }|{p,q}\rangle e^{-i(p+q)\widetilde{\Phi } \left( t\right) }e^{\beta \frac{\mathscr {C}}{4}} e^{-\frac{1}{4}\left( k+l-k'-l'\right) ^2\Gamma \left( t\right) }, \end{aligned}$$

where $\mathscr {C} = \sum _r \frac{4|{g_r}|^2}{\omega _r}$. Using these results, the two-qubit density matrix takes the form

27 $$\begin{aligned} \left[ \rho _S \left( t\right) \right] _{k',l';k,l} =\left[ \rho _S \left( 0\right) \right] _{k',l';k,l}X \left( t\right) e^{-i\frac{\omega _0}{2} \left( k'+l'-k-l\right) t} e^{-i\frac{\Delta \left( t\right) }{2} \left( k'l'-kl\right) } e^{-\frac{1}{4}\left( k+l-k'-l'\right) ^2\Gamma \left( t\right) }, \end{aligned}$$

where the initial state $\left[ \rho _S \left( 0\right) \right] _{k',l';k,l} =\langle {k',l'}||{\psi }\rangle \langle {\psi }| |{k,l}\rangle$, while

$$\begin{aligned} X\left( t\right) =\frac{\sum _{p,q}e^{-\frac{\beta \omega _0}{2} \left( p+q\right) }|{\langle {p,q}|{\psi }\rangle }|^2e^{\beta (p+q)^2 \frac{\mathscr {C}}{4}}e^{-i(p+q)\widetilde{\Phi }\left( t\right) }}{\sum _{p,q}e^{-\frac{\beta \omega _0}{2}\left( p+q\right) } |{\langle {p,q}|{\psi }\rangle }|^2e^{\beta (p+q)^2 \frac{\mathscr {C}}{4}}}. \end{aligned}$$

## Data Availability

The datasets used and/or analyzed during the current study are available from the corresponding author on reasonable request.
